# Preclinical investigations and first-in-human application of ^152^Tb-PSMA-617 for PET/CT imaging of prostate cancer

**DOI:** 10.1186/s13550-019-0538-1

**Published:** 2019-07-25

**Authors:** Cristina Müller, Aviral Singh, Christoph A. Umbricht, Harshad R. Kulkarni, Karl Johnston, Martina Benešová, Stefan Senftleben, Dirk Müller, Christiaan Vermeulen, Roger Schibli, Ulli Köster, Nicholas P. van der Meulen, Richard P. Baum

**Affiliations:** 1 Center for Radiopharmaceutical Sciences ETH-PSI-USZ, Paul Scherrer Institute, 5232 Villigen-PSI, Switzerland; 2 Theranostics Center for Molecular Radiotherapy and Precision Oncology, ENETS Center of Excellence, Zentralklinik Bad Berka, Robert-Koch-Allee 9, 99437 Bad Berka, Germany; 3 GROW - School for Oncology and Developmental Biology, Maastricht University, Maastricht, Netherlands; 4 CERN, Geneva, Switzerland; 5 Department of Chemistry and Applied Biosciences, ETH Zurich, Zurich, Switzerland; 6 Institut Laue-Langevin, Grenoble, France; 7 Laboratory of Radiochemistry, Paul Scherrer Institute, Villigen-PSI, Switzerland

**Keywords:** ^152^Tb, Terbium, PSMA-617, PET/CT imaging, Theragnostics, Prostate cancer

## Abstract

**Background:**

For almost a decade, terbium radioisotopes have been explored for their potential theragnostic application in nuclear medicine: ^152^Tb and ^155^Tb are the radioisotopes identified for PET or SPECT imaging, while ^149^Tb and ^161^Tb have suitable decay characteristics for α- and combined β^−^/Auger-e^−^-therapy, respectively. In the present study, the application of ^152^Tb, in combination with PSMA-617 for imaging of prostate-specific membrane antigen (PSMA)-positive prostate cancer, was demonstrated in a preclinical setting and in a patient with metastatic castration-resistant prostate cancer (mCRPC).

**Results:**

^152^Tb was produced at the ISOLDE facility at CERN/Geneva, Switzerland, by spallation, followed by on-line mass separation. The chemical separation was performed at Paul Scherrer Institute using chromatographic methods, as previously reported. ^152^Tb was employed for labeling PSMA-617, and the radioligand was extensively investigated in vitro to demonstrate similar characteristics to its ^177^Lu-labeled counterpart. Preclinical PET/CT imaging studies performed with mice enabled visualization of PSMA-positive PC-3 PIP tumors, while uptake in PSMA-negative PC-3 flu tumors were absent. Based on these promising preclinical results, ^152^Tb was shipped to Zentralklinik Bad Berka, Germany, where it was used for labeling of PSMA-617, enabling PET imaging of a patient with mCRPC. PET/CT scans were performed over a period of 25 h post injection (p.i.) of the radioligand (140 MBq). The images were of diagnostic quality, particularly those acquired at later time points, and enabled the detection of the same metastatic lesions and of local recurrent tumor as previously detected by ^68^Ga-PSMA-11 PET/CT acquired 45 min p.i.

**Conclusions:**

The results of this study demonstrate the successful preparation and preclinical testing of ^152^Tb-PSMA-617 and its first application in a patient with mCRPC. This work could pave the way towards clinical application of other Tb radionuclides in the near future, most importantly ^161^Tb, which has promising decay characteristics for an effective treatment of mCRPC patients.

**Electronic supplementary material:**

The online version of this article (10.1186/s13550-019-0538-1) contains supplementary material, which is available to authorized users.

## Background

Terbium (Tb) radioisotopes have been proposed for medical applications several years ago [1, 2]. Four Tb isotopes are of interest in this regard: ^152^Tb and ^155^Tb can be used for nuclear imaging, whereas ^149^Tb and ^161^Tb have suitable decay characteristics for targeted radionuclide therapy [3]. Tb radioisotopes can be stably coordinated using the 1,4,7,10-tetraazacyclododecane-1,4,7,10-tetraacetic acid (DOTA) chelator, which is applied in a series of clinically employed radiopharmaceuticals [3–8]. Based on these circumstances, the preparation of chemically identical radiopharmaceuticals for diagnosis and therapy is feasible and would allow the realization of the radiotheranostic concept [3]. ^152^Tb is a β^+^-emitter that can be used for positron emission tomography (PET), whereas ^155^Tb emits γ-radiation suitable for single-photon emission computed tomography (SPECT). Both radioisotopes were employed preclinically to demonstrate their potential for tumor imaging with a variety of targeting ligands [3, 5, 7]. The relatively long half-lives of these radionuclides (^152^Tb: 17.5 h and ^155^Tb: 5.32 days) may facilitate their application for pre-therapeutic dosimetry or allow their use in combination with longer-lived biomolecules (e.g., antibodies or albumin-binding small molecules) that require imaging at late time points after injection.

^161^Tb is gaining increasing attention among the radiopharmaceutical and nuclear medical community due to its interesting decay characteristics for therapeutic purposes and the feasibility of producing this radionuclide in high quantities and quality [4]. ^161^Tb (T_1/2_ = 6.89 days; Eβ^−^_average_ = 154 keV) is characterized by decay properties that are similar to those of ^177^Lu (T_1/2_ = 6.65 d, Eβ^−^_average_ = 134 keV) which is currently the most successfully employed therapeutic radiometal in nuclear oncology. In addition to the β^−^-particles, ^161^Tb also emits a substantial number of conversion and conversion and Auger-e^−^ [4]. It is believed that the high multiplicity and high linear energy transfer (LET) of the low-energy electrons emitted by ^161^Tb would result in effective killing of single cancer (stem) cells and cancer cell clusters. A number of theoretical dosimetry studies performed over the years consistently predicted the high potential of this radionuclide for nuclear oncology purposes [9–11]. Preclinical experiments performed with ^161^Tb-folate demonstrated that the conversion and Auger-e^−^ are beneficial with regard to the tumor treatment, while additional side effects to the kidneys were not observed when compared to the effects observed with ^177^Lu-folate [6, 12].

^149^Tb is an α-emitter and, hence, belongs to the group of radionuclides which currently has the nuclear medicine physicians’ undivided attention. The positive experience with ^225^Ac- and ^213^Bi-labeled small molecules for targeting the prostate-specific membrane antigen (PSMA), as well as the introduction of ^223^RaCl_2_ (Xofigo™) for the treatment of prostate cancer-related bone metastases, have escalated the concept of α-therapy. With a half-life of 4.12 h, ^149^Tb is much shorter-lived than ^225^Ac (*T*_1/2_ = 10.0 days), but still provides a more convenient lifespan as compared to ^213^Bi (T_1/2_ = 46 min). Almost two decades ago, Beyer et al. performed a study with ^149^Tb-labeled rituximab which prevented progression of the disease in a mouse model of leukemia [2]. Later, ^149^Tb was also used in combination with a DOTA-folate conjugate and applied in preclinical therapy studies in which a dose-dependent tumor growth inhibition was demonstrated [13]. The short half-life of ^149^Tb may make it particularly interesting in combination with small tumor-targeting molecules that are characterized by fast accumulation in the tumor tissue and efficient clearance from healthy organs and tissues. An interesting characteristic of ^149^Tb is the emission of β^+^-particles (Eβ^+^ = 730 keV; *I* = 7.1%), potentially enabling PET imaging as recently exemplified in a preclinical study, in which the concept of alpha-PET was proposed [8]. Clinical application will, however, only be possible once the challenge of producing this radionuclide has been addressed by the construction of the required production facilities.

^152^Tb has been the first of the four radioisotopes to be applied clinically, using ^152^Tb-DOTATOC in a patient with metastatic well-differentiated functional neuroendocrine neoplasm of the ileum [14]. The images were convincing and, owing to the relatively long half-life of ^152^Tb, scanning over an extended time period was feasible. This enabled easy visualization of metastases in spite of the unfavorably high energy and low intensity of the emitted β^+^-particles (Eβ^+^_average_ = 1140 keV; *I* = 20%) [14].

In this study, it was aimed to take another step towards clinical application of terbium radioisotopes in combination with a PSMA-targeting agent for future radiotheragnostics of prostate cancer. As a result, we used ^152^Tb for the labeling of a PSMA-targeting ligand, PSMA-617, which is currently being studied in a phase III clinical trial (NCT03511664, Endocyte, USA), in combination with ^177^Lu, for targeted radionuclide therapy of metastasized castration-resistant prostate cancer (mCRPC). ^152^Tb-PSMA-617 was extensively investigated in vitro and applied to tumor-bearing mice for PET imaging studies. Based on promising preclinical data, PSMA-617 was labeled with ^152^Tb at Zentralklinik Bad Berka, Germany, and used for PET/CT imaging of a mCRPC patient. The results of this study paved the path towards a potential clinical application of ^161^Tb as the therapeutic counterpart with high potential to be effectively applied for the treatment of prostate cancer.

## Methods

### Synthesis of ^152^Tb-PSMA-617 for preclinical studies

^152^Tb was produced by proton-induced spallation in a tantalum target, followed by ionization of the spallation products and mass separation at ISOLDE (CERN, Geneva, Switzerland), as previously reported [7]. In short, mass 152 isobars were implanted into zinc-coated gold foils and the collected samples were left to stand, to allow short-lived radionuclides to decay before transportation to Paul Scherrer Institute (PSI), Switzerland. The zinc was dissolved from the gold foil using dilute ammonium nitrate, and the isobars loaded onto a macroporous strongly acidic cation exchange resin (Sykam Vertriebs GmbH, Germany). ^152^Tb was separated from impurities using gradient elution with α-hydroxy-isobutyric acid (α-HIBA, pH 4.7) [7].

PSMA-617 (Advanced Biochemical Compounds, ABX GmbH, Germany) was dissolved in MilliQ water (1 mM). The labeling of PSMA-617 was performed directly in the ^152^Tb solution consisting of α-HIBA (pH 4.7) as it was obtained from the separation process. The reaction mixture was prepared by means of addition of PSMA-617 (1 mM stock solution) to the ^152^Tb-containing α-HIBA solution to obtain the desired molar activity and incubated for 15 min at 95 °C. Quality control was performed by HPLC (Additional file 1). ^152^Tb-PSMA-617 was diluted with saline and used for the individual experiments without further purification.

No-carrier-added ^177^Lu was obtained from Isotope Technologies Garching GmbH (ITG GmbH, Germany. The radiolabeling of PSMA-617 with ^177^Lu was performed as previously reported [15].

### Preclinical in vitro evaluation of ^152^Tb-PSMA-617

The stability of ^152^Tb-PSMA-617 (120 MBq in 3 mL PBS; 10 MBq/nmol) was investigated by incubation of the radioligand solution with and without addition of 3 mg L ascorbic acid. Quality control was performed using HPLC after 2 h, 16 h, 40 h, and 90 h incubation time at room temperature, respectively (Additional file 1).

Cell uptake and internalization of ^152^Tb-PSMA-617 and ^177^Lu-PSMA-617 was performed using PSMA-positive PC-3 PIP and PSMA-negative PC-3 flu cells (kindly provided by Martin Pomper, Johns Hopkins University, Baltimore, USA) after 1 h, 2 h, 4 h, and 6 h incubation time, respectively, as previously reported (Additional file 1) [15].

### Preclinical in vivo evaluation of ^152^Tb-PSMA-617

#### Tumor mouse model

In vivo experiments were approved by the local veterinary department and conducted in accordance with the Swiss law of animal protection. Female, athymic BALB/c nude mice were obtained from Charles River Laboratories (Sulzfeld, Germany) at the age of 5–6 weeks. PC-3 PIP cells (6 × 10^6^ tumor cells) and PC-3 flu cells (5 × 10^6^ tumor cells) were subcutaneously injected in 100 μL Hank’s balanced salt solution (HBSS containing Ca^2+^/Mg^2+^) on the right and left shoulders, respectively, of the mice on the same day, 12–14 days before performing the in vivo experiments.

#### Evaluation of tumor-to-organ ratios

^152^Tb-PSMA-617 (5 MBq, 1 nmol, 100 μL) was injected intravenously to athymic BALB/c nude mice with PC-3 PIP and PC-3 flu xenografts. Mice were sacrificed at 1 h, 4 h, and 24 h post injection (p.i.) of the radioligand. The blood, tumors, liver, and kidneys were collected and weighed before counting for radioactivity using a γ-counter (Perkin Elmer, Wallac Wizard 1480). The results were decay-corrected and listed as percentage injected activity per gram of tissue mass (% IA/g) to calculate the tumor-to-blood, tumor-to-liver, and tumor-to-kidney ratios.

#### PET/CT and SPECT/CT imaging studies

PET/CT scans were performed using a small-animal bench-top PET/CT scanner (G8, Perkin Elmer, MA, USA; Additional file 1). Mice were injected intravenously with ^152^Tb-PSMA-617 (10 MBq, 1 nmol, 100 μL, diluted in saline) and anesthetized with a mixture of isoflurane and oxygen for in vivo scans (PET/CT and SPECT/CT). Static whole-body PET scans, 10 min in duration, were performed at 2 and 15 h p.i. of the radioligand, followed by a CT scan of 1.5 min. SPECT/CT studies were performed using a small-animal SPECT/CT scanner (NanoSPECT/CTTM, Mediso Medical Imaging Systems, Budapest, Hungary) (Additional file 1). Tumor-bearing mice were intravenously injected with ^177^Lu-PSMA-617 (25 MBq, 1 nmol, 100 μL, diluted in saline). Static SPECT/CT scans, 45 min in duration, were performed at 2 h and 15 h p.i. of the radioligand, followed by a CT scan of 7.5 min. Reconstruction of the acquired data was performed using the software of the scanner in question. All images were prepared using *VivoQuant* post-processing software (version 3.5, inviCRO Imaging Services and Software, Boston, USA). A Gauss post-reconstruction filter (full width at half maximum = 1 mm) was applied to the images and the scale adjusted by cutting 5% of the lower signal intensity to make the tumors and kidneys readily visible.

### Radioligand preparation for clinical application

A solution of ^152^Tb in α-HIBA was transported from PSI to Zentralklinik Bad Berka (ZBB). ^152^Tb was used for the radiolabeling of PSMA-617 (Advanced Biochemical Compounds, ABX GmbH, Germany). In brief, PSMA-617 (40 μg in 1 mL MilliQ water) was labeled with 169 MBq ^152^Tb (α-HIBA, 1 mL; 0.11 M) at pH 5. The reaction mixture was incubated at 95 °C for 20 min. Quality control was performed using analytical HPLC (Jasco PU-1580 system) equipped with a radiometric detector and a reversed-phase column (Jupiter™ Proteo 90 Å, LC, C-18, 4 μm, 250 × 4.6 mm, Phenomenex). The mobile phase consisted of MilliQ water containing 5% acetonitrile and 0.1% trifluoroacetic acid (A) and acetonitrile containing 0.5% MilliQ water and 0.1% trifluoroacetic acid (B). The gradient from 100% A to 100% B over a period of 15 min was used at a flow rate of 1 mL/min. The reaction solution was diluted with 3 mL sterile saline (NaCl 0.9%) and filtered using a 0.2-μm sterile filter. Samples were taken for sterility and endotoxin testing using an Endosafe®-PTS™ cartridge. The pH value of the final product was determined using pH strips.

### Ethical and regulatory issues

^152^Tb-PSMA-617 was administered in compliance with the German Medicinal Products Act (section 13, subsection 2b) and the 1964 Declaration of Helsinki. The study was approved by an institutional review board and the patient signed written informed consent prior to the investigation, which was performed in accordance with the regulations of the German Federal Agency for Radiation Protection. Additionally, written informed consent was obtained by the patient for collection and storage of his data in the institutional electronic databank, as well as the publication of the said data.

### Patient selection and characteristics

A 59-year-old patient suffering from poorly differentiated, hormone-refractory prostate adenocarcinoma with residual primary tumor infiltrating both seminal vesicles, multiple lymph node, and bone metastases was selected for the study (Table 1). At initial diagnosis, he had metastatic disease (stage IV) and a Gleason score of 8 (4 + 4). At the time of the study, his general status was good (Karnofsky Performance Score 90%): he did not complain of serious symptoms and had no significant associated diseases. He presented for whole body restaging under androgen deprivation therapy with Leuprorelin (Trenantone™), an analog of the gonadotropin-releasing hormone (GnRH), to evaluate the possibility of performing radioligand therapy with ^177^Lu-PSMA-617.

**Table 1:** Characteristics of the patient

Characteristics	Patient
Age	59 years
Gender	Male
Height	186 cm
Body weight	90 kg
Cancer type	Metastatic, hormone-refractory, poorly-differentiated prostate adenocarcinoma
Gleason score	8 (4 + 4)
Metastases	Residual primary tumor within the prostate bed with bilateral seminal vesicle infiltration and multiple lymph node as well as bone metastases
Stage of the disease (at initial diagnosis)	IV
Karnofsky index (at the time of this study)	90%
Relevant previous surgery	Cytoreductive Da-Vinci assisted robotic prostatovesiculectomy with bilateral regional lymphadenectomy
Other relevant treatment	Androgen deprivation therapy with NSAA (Bicalutamide) and GnRH analogue (Leuprorelin, Trenantone™)
PET/CT scan (^152^Tb-PSMA-617)	24 Days after PET/CT scan with ^68^Ga-PSMA-11

*NSAA* non-steroidal antiandrogen, *GnRH* gonadotropin-releasing hormone

### Clinical PET/CT imaging and reconstruction parameters

PET/CT imaging was performed using a Biograph mCT Flow 64 scanner from Siemens Healthcare AG. Whole-body PET/CT scans were acquired in the supine position from vertex to feet at various time intervals. PET scans were obtained using the continuous bed motion mode with a speed of 1.5 mm/s for the acquisitions 50 min and 2 h p.i. The 18.5 h and 25 h scans were acquired using a scan speed of 1.1 mm/s. Reconstructions were performed using recon TrueX+ToF with 3 iterations and 21 subsets and images were prepared using a Gaussian filter (2 mm FWHM) and a matrix size of 200. CT scans were acquired at 100 kV and at pitch of 1.5. CareDose4D was applied using a 0.5-s gantry rotation time, 5-mm slice thickness, and a matrix size of 512 × 512.

### Clinical PET/CT imaging using ^152^Tb-PSMA-617

^152^Tb-PSMA-617 (140 MBq) was administered to the patient as intravenous bolus into a peripheral arm vein. This quantity of activity was chosen to enable scanning over an extended period after application. After injection, 4 sets of PET/CT images were acquired at 50 min (standard acquisition time point), at 2 h (moderately delayed scan time), and at 18.5 h and 25 h p.i. (delayed scan time), respectively. Low-dose CT was performed for attenuation correction after oral administration of Mucofalk™ (1 L), a negative intestinal contrast agent.

### Image analysis

The ^152^Tb-PSMA-617 PET/CT images were interpreted independently by two experienced physicians (two board-certified nuclear medicine physicians, each with over 10 years of experience in reporting PET/CT studies). A qualitative evaluation of the scans was performed visually by analyzing the PSMA-avid lesions on the transverse, coronal, and sagittal sections as well as visually on the maximum intensity projection (MIP) images. The PET/CT images obtained with ^152^Tb-PSMA-617 were compared with those obtained 1 month earlier using ^68^Ga-PSMA-11. This allowed a comparison of the feasibility of PET/CT imaging using ^152^Tb-PSMA-617 as compared to PSMA-11 for restaging of the disease.

## Results

### Chemical separation of ^152^Tb

^152^Tb was successfully separated from other mass 152 isobar impurities, producing a radionuclidically pure product. Up to 600 MBq ^152^Tb was eluted and used for radiolabeling purposes to conduct preclinical experiments at PSI as well, as for the delivery of the radionuclide to ZBB, Germany, where the radiolabeling was performed for patient application.

### Preclinical investigations

#### Preparation of ^152^Tb-PSMA-617

The labeling of PSMA-617 with ^152^Tb resulted in radiochemically pure radioligand (> 98%) up to specific activities of 20 MBq/nmol (Additional file 1: Figure S1). ^152^Tb-PSMA-617 was stable in PBS over at least 90 h (> 95%) without the addition of l-ascorbic acid (Additional file 1: Table S1).

#### Cell uptake and internalization

**Fig. 1:**
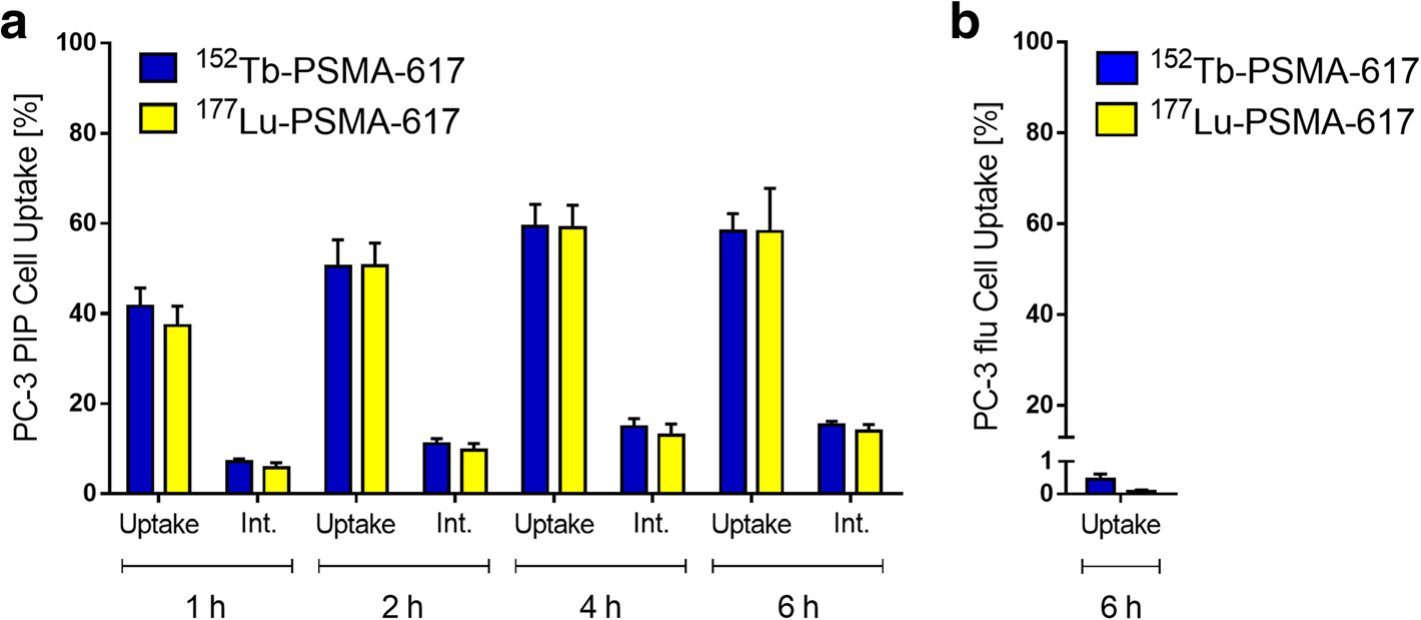
Uptake and internalization of ^152^Tb-PSMA-617 (blue) compared to ^177^Lu-PSMA-617 (yellow) in **a** PSMA-positive PC-3 PIP tumor cells and **b** PSMA-negative PC-3 flu tumor cells

PSMA-specific uptake and internalization of ^152^Tb-PSMA-617 and ^177^Lu-PSMA-617, investigated using PC-3 PIP and PC-3 flu tumor cells, showed equal results for both radioligands (Fig. 1). The uptake of ^152^Tb-PSMA-617 and ^177^Lu-PSMA-617 into PC-3 PIP tumor cells was high (38–42% of total added activity) already after 1 h and increased to > 58% after 6 h-incubation time. A fraction of 6–7% of total added activity was internalized after 1 h-incubation, which increased to 14–15% after 6 h-incubation time (Fig. 1a). The uptake of ^152^Tb-PSMA-617 and ^177^Lu-PSMA-617 was < 1% for PC-3 flu tumor cells, which proved that the uptake in PC-3 PIP tumor cells was PSMA-specific (Fig. 1b).

#### In vivo evaluation in tumor-bearing mice

Tumor-to-organ ratios of ^152^Tb-PSMA-617 were evaluated in a PC-3 PIP/flu xenograft mouse model over a period of 24 h and compared to the values previously obtained with ^177^Lu-PSMA-617 [16]. ^152^Tb-PSMA-617 showed, relative to ^177^Lu-PSMA-617, slightly lower tumor-to-liver ratio, but increased tumor-to-blood and tumor-to-kidney ratios at early time points (1 h p.i.). These values were increased at later time points, resulting in tumor-to-blood and tumor-to-liver ratios > 200 at 24 h p.i. in both cases, while tumor-to-kidney ratios were in the same range for both radioligands (Table 2).

**Table 2:** Biodistribution of ^152^Tb-PSMA-617 in PC-3 PIP/flu tumor-bearing mice

	^152^Tb-PSMA-617			^177^Lu-PSMA-617*		
	1 h p.i.	4 h p.i.	24 h p.i.	1 h p.i.	4 h p.i.	24 h p.i.
Tumor-to-blood	> 200	> 200	> 200	88 ± 16	> 200	> 200
Tumor-to-liver	159 ± 20	179 ± 13	> 200	> 200	> 200	> 200
Tumor-to-kidney	5.7 ± 0.5	19 ± 3	53 ± 19	4.5 ± 0.8	16 ± 3	50 ± 5

*Data obtained from Benešová et al. [16]

**Fig. 2:**
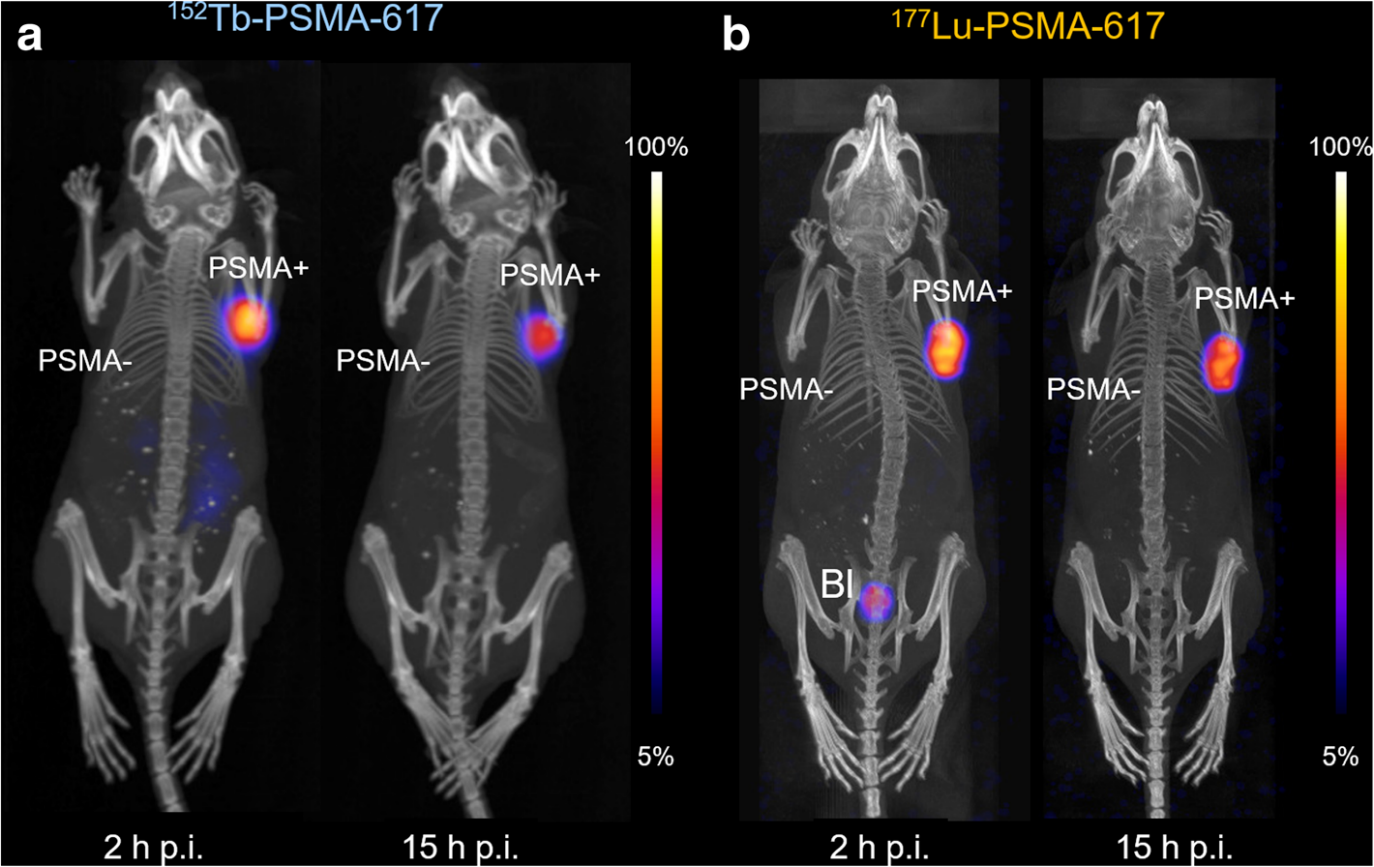
Nuclear images of PC-3 PIP/flu tumor-bearing mice at 2 h and 15 h post injection of **a** ^152^Tb-PSMA-617 (PET/CT) and **b** ^177^Lu-PSMA-617 (SPECT/CT). PSMA+, PSMA-positive PC-3 PIP tumor; PSMA-, PSMA-negative PC-3 flu tumor; Bl, urinary bladder

PET/CT and SPECT/CT scans were performed with PC-3 PIP/flu tumor-bearing mice at 2 h and 15 h after injection of ^152^Tb-PSMA-617 and ^177^Lu-PSMA-617, respectively. Uptake of activity was already detectable in the PC-3 PIP tumors 2 h after injection of either radioligand. Only marginal amounts of activity were detectable in the kidneys for ^152^Tb-PSMA-617 and ^177^Lu-PSMA-617 at 2 h p.i., with complete wash-out at 15 h p.i. No accumulation of radioligand was observed in the PC-3 flu tumors on the left shoulder or any other non-target organ, thus indicating PSMA-specific tumor uptake (Fig. 2).

### First-in-human application

#### Radioligand preparation for patient application

The radiochemical purity of the ^152^Tb-PSMA-617, prepared at ZBB, was > 99%, which allowed its application without any further purification. The pH value of the final product was 4.6, while the bacterial endotoxin test was negative. Due to the small application volume (< 3 mL), adjustment of the osmolarity of the injection solution was not necessary.

#### Physiological and pathological uptake

**Fig. 3:**
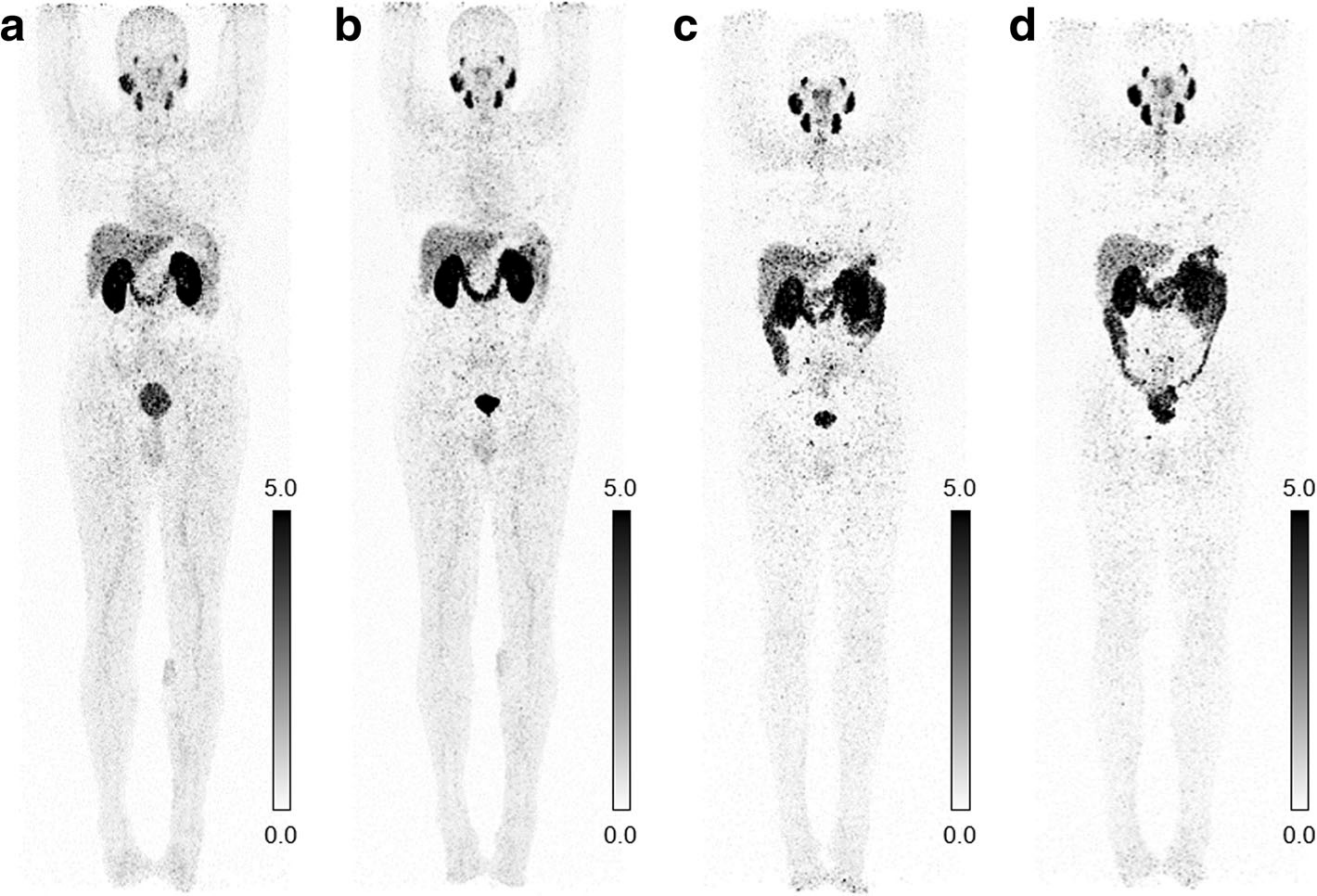
Physiological biodistribution of ^152^Tb-PSMA-617 on PET/CT scans (MIP images) obtained at **a** 50 min, **b** 2 h, **c** 18.5 h, and **d** 25 h, respectively, after injection of 140 MBq ^152^Tb-PSMA-617. Physiological accumulation of the radioligand is observed in the blood pool (decreasing over time), lacrimal, parotid, and submandibular glands (strong), the nasopharyngeal mucosa as well as in the liver, spleen (faint), and the intestinal tract (increasing over time). Uptake in the kidneys and retention of activity in the urinary bladder are due to renal excretion of ^152^Tb-PSMA-617. Mild background activity of the radioligand was observed in normal tissue which decreased over time

Early ^152^Tb-PSMA-617 PET/CT images demonstrated normal blood pool activity, including the heart and the blood vessels. Mild background activity of the radioligand was observed in normal tissue which decreased over time. Moderate activity accumulation was visualized in the liver, whereas only mild radioligand uptake was seen in the spleen (Fig. 3). Uptake and retention of activity in the kidneys and in the urinary bladder were due to renal excretion of ^152^Tb-PSMA-617. High accumulation of ^152^Tb-PSMA-617 was visualized in the lacrimal, parotid, and submandibular glands, as well as in the nasopharyngeal mucosa and the intestinal tract (significantly increasing over time), as is the case when using ^177^Lu-PSMA-617.

**Fig. 4:**
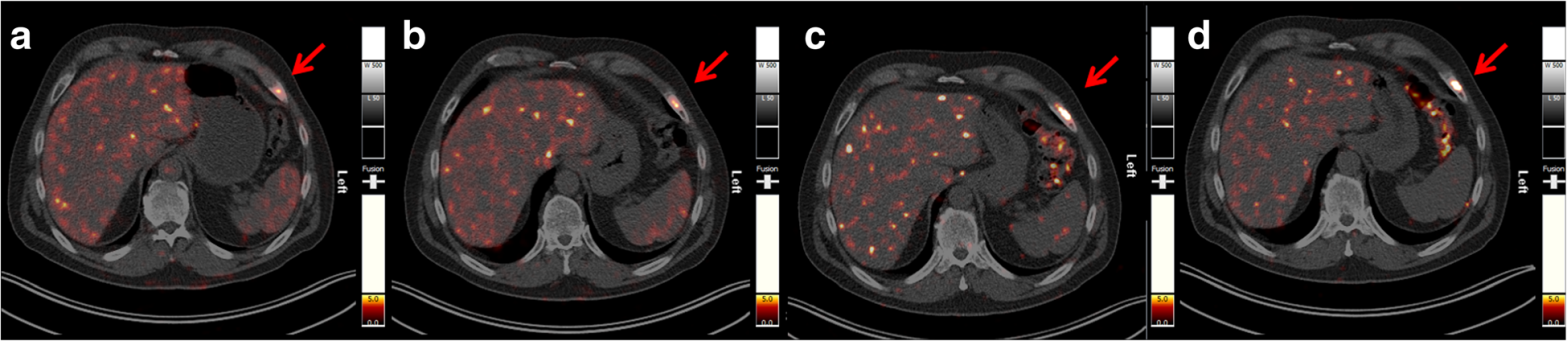
PET/CT scans (transversal slices through the upper abdomen at the level of the liver and spleen) obtained at **a** 50 min, **b** 2 h, **c** 18.5 h, and **d** 25 h, respectively after injection of 140 MBq ^152^Tb-PSMA-617, clearly demonstrating a PSMA-avid bone metastasis in the ventrolateral part of the left 7th rib (red arrow) with maximum uptake at 18.5 and 25 h p.i.

**Fig. 5:**
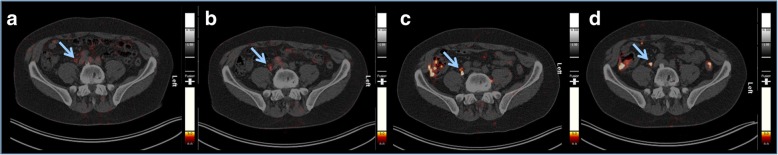
PET/CT scans (transversal slices through the lower abdomen at the entrance level of the pelvis) obtained at **a** 50 min, **b** 2 h, **c** 18.5 h, and **d** 25 h, respectively after injection of 140 MBq ^152^Tb-PSMA-617, revealing a small lymph node metastasis (< 8 mm in size) near the right common iliac artery (blue arrow) with maximum uptake observed at 18.5 and 25 h p.i.

The PET/CT images demonstrated several metastatic lesions in this patient. The optimal image contrast for the detection of metastases was obtained 18.5 and 25 h after administration of ^152^Tb-PSMA-617 (Figs. 4 and 5).

**Fig. 6:**
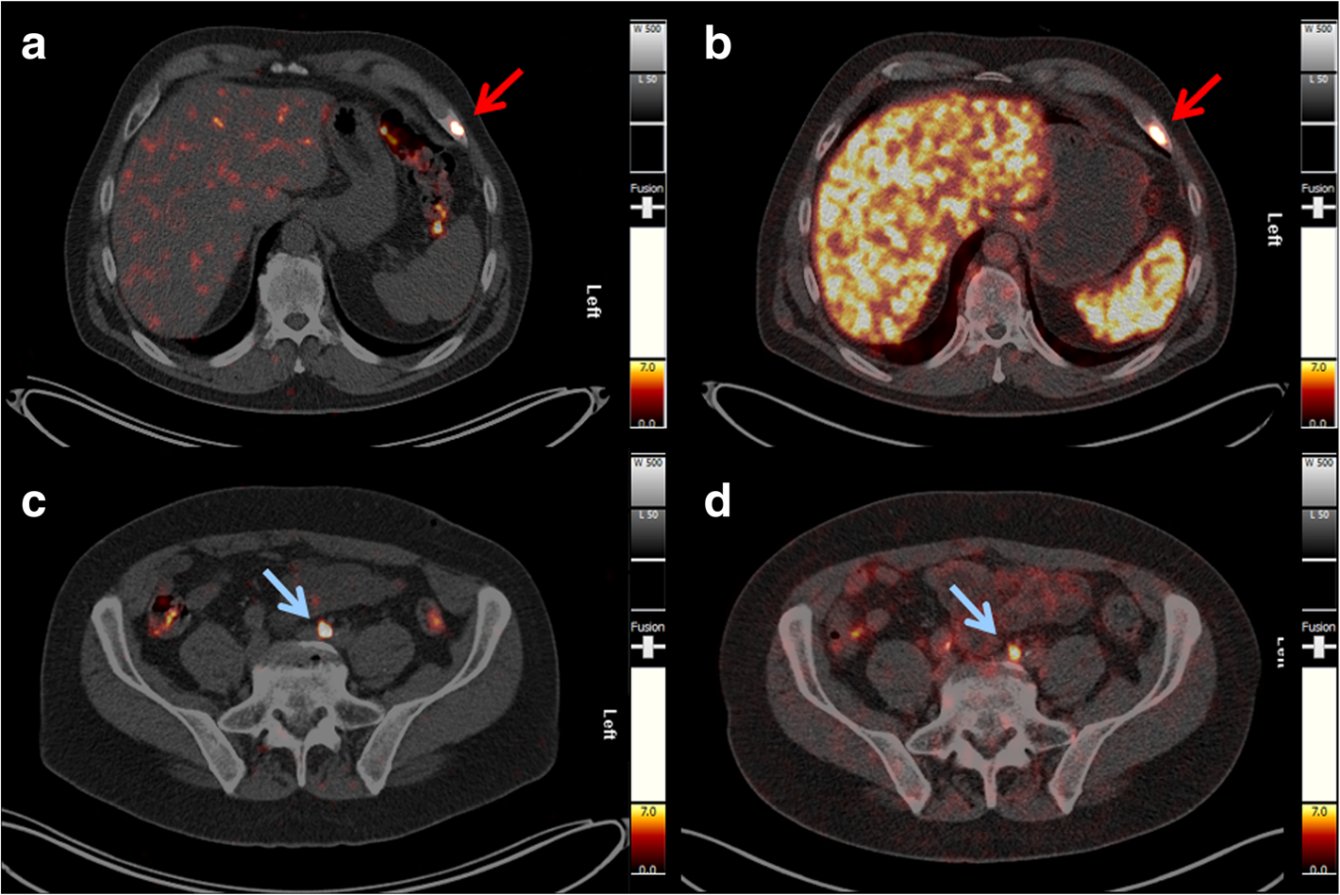
Comparison of PET/CT scans obtained with ^152^Tb-PSMA-617 and ^68^Ga-PSMA-11, respectively. **a**, **c** PET/CT image acquired 18.5 h after injection of 140 MBq of ^152^Tb-PSMA-617. **b**, **d** PET/CT image acquired 45 min after injection of 160 MBq of ^68^Ga-PSMA-11. Red arrows show a bone metastasis in the ventrolateral part of the left 7th rib, and blue arrows show a small lymph node metastasis along the left common iliac artery

The PET/CT images acquired with ^152^Tb-PSMA-617 were compared with those obtained with ^68^Ga-PSMA-11 1 month earlier: all lymph node and bone metastases, as well as residual/recurrent disease in the seminal vesicles, were detected identically with both radioligands (Fig. 6).

#### Clinical safety of ^152^Tb-PSMA-617

The imaging procedure was well tolerated by the patient. No adverse effects such as nausea, emesis, rash, erythema, pruritus or fever were observed or reported by the patient during, immediately after, or at follow-up checks of the patient after administration of ^152^Tb-PSMA-617. According to the Common Terminology Criteria for Adverse Events (CTCAE v5.0), there were no significant changes in the relevant laboratory values (hematological, renal, and hepatic panel) over 6 months follow up of the patient after the administration of ^152^Tb-PSMA-617 (Table 3).

**Table 3:** Laboratory parameters of the patient before and after ^152^Tb-PSMA-617 administration

Parameters	Normal range	Units	Patient	
			Before	After
Hemoglobin	8.6–12.1	mmol/L	9.0	9.5
Leukocytes	4.3–10	Gpt/L	5.9	5.1
Thrombocytes	150–400	Gpt/L	194	207
Urea	3.0–9.2	mmol/L	5.2	4.8
Creatinine	62–106	μmol/L	62.0	67.1
eGFR (MDRD)	> 60	mL/min/1.73m^2^	> 60	> 60
ALT	< 0.83	μmol/s/L	0.37	0.27
AST	< 0.85	μmol/s/L	0.36	0.27
GGT	0.17–1.19	μmol/s/L	0.70	0.44

*eGFR* (MDRD) estimated glomerular filtration rate, *ALT* alanine transaminase, *AST* aspartate transaminase, *GGT* gamma-glutamyl transpeptidase, *Before* 1 month before the PET/CT scan performed with ^152^Tb-PSMA-617, *After* at the time of the next restaging, 6 months after the ^152^Tb-PSMA-617 application

## Discussion

Terbium offers four radioisotopes that potentially allow the production of radiopharmaceuticals with identical chemical properties to be used for all modalities in nuclear medicine, namely, PET and SPECT imaging as well as α- and β^−^/Auger-e^−^ radionuclide therapy [3]. In the present study, we investigated ^152^Tb, which is a positron emitter and, hence, useful for PET imaging purposes. PSMA-617 was labeled with ^152^Tb and preclinically evaluated. In line with previous results that proved that ^152/161^Tb and ^177^Lu are interchangeable without affecting the radioligand’s chemical and pharmacokinetic properties [6, 7], our data demonstrated similar behavior of ^152^Tb-PSMA-617 and ^177^Lu-PSMA-617 with regard to PSMA-specific cell uptake and internalization. PET scans performed with tumor-bearing mice injected with ^152^Tb-PSMA-617 revealed pharmacokinetic properties as expected: high activity uptake was observed in PSMA-positive PC-3 PIP tumors, but negligible accumulation in PSMA-negative PC-3 flu tumors, as previously also shown with ^177^Lu-PSMA-617 [15]. These findings were in agreement with recent investigations that proved the same in vivo behavior of ^161^Tb-PSMA-617 and ^177^Lu-PSMA-617, respectively [17].

The clinical PET/CT images obtained with ^152^Tb-PSMA-617 in a patient with metastatic prostate cancer were of diagnostic quality, enabling the visualization of all target lesions previously detected with ^68^Ga-PSMA-11, including the clear identification of specific radioligand uptake in bone and lymph node metastases as well as in recurrent disease in the seminal vesicles. Given the abovementioned physical properties of ^152^Tb, the acquired images were somewhat noisy, which could result in the identification of tiny false-positive lesions by inexperienced investigators, especially if the necessary correlations with anatomic imaging (CT) are not performed carefully. In this patient, however, the previously performed ^68^Ga-PSMA-11 PET/CT was also available. In the future, significant improvement of image quality can be expected by applying dedicated software updates specific for ^152^Tb-based PET imaging. The background activity in soft tissues cleared effectively over time; however, there was significant excretion of radioligand in the intestines, which could render the detection of small peritoneal metastases difficult. It is worthy to note that the background activity in the liver was very low on late images compared to that of the corresponding ^68^Ga-PSMA-11 PET/CT scan acquired 45 min p.i. The higher target-to-background ratio at later time points after injection of ^152^Tb-PSMA-617 may be favorable to diagnose liver lesions with more confidence compared to what is feasible based on conventional ^68^Ga-PSMA-11 images. The promising preclinical data of ^152^Tb-PSMA-617, which were similar to those obtained with ^177^Lu-PSMA-617, and the impressive patient images at late time points after injection give rise to the assumption that ^152^Tb-PSMA-617 would be of great value for pre-therapeutic dosimetry and, therefore, could play an important role in therapy planning. On the other hand, a long half-life is a disadvantage for diagnostic imaging where scanning early after injection is of interest for efficient logistics at a hospital. In this case, the slightly increased estimated dose delivered to normal organs and tissue, when using ^152^Tb instead of ^68^Ga, would be a disadvantage. It is not of concern, however, for pre-therapeutic application since the additionally absorbed dose would be only a negligible fraction of the dose delivered by therapeutic quantities of ^177^Lu-PSMA-617.

Despite the fact that proton-induced spallation repeatedly yielded ^152^Tb in suitable amounts for preclinical studies, the current production capabilities will not allow making ^152^Tb available at the larger quantities that would be necessary for the introduction of this radionuclide into clinical studies. We are, however, convinced that—in view of a clinical application—^155^Tb will be the diagnostic Tb radioisotope of choice due to the more favorable possibilities of production routes. ^155^Tb could be produced at a medical cyclotron using the ^155^Gd(p,n)^155^Tb nuclear reaction, potentially enabling the production of GBq-quantities sufficient for clinical translation. Although PET/CT is still the favored method over SPECT/CT, due to its higher sensitivity and resolution, continuous progress in SPECT/CT scanners (including quantification similar to SUV) will enable also the use of SPECT radioisotopes. As the first radiometal-based theragnostic pair for clinical application, ^155^Tb may be suitable for personalized pre-therapeutic imaging and dosimetry prior to therapy with its ^161^Tb-labeled counterpart.

## Conclusion

In this study, we demonstrated the similar pharmacokinetic properties of ^152^Tb-PSMA-617 and ^177^Lu-PSMA-617 on a preclinical level. Clinically, it was shown in a prostate cancer patient that ^152^Tb-PSMA-617 was safe and enabled detection of all bone and lymph node metastases, as well as of regional recurrent disease initially detected with ^68^Ga-PSMA-11 PET/CT one month earlier. The image quality of the scans recorded with ^152^Tb-PSMA-617 was not as high as those obtained with ^68^Ga-PSMA-11; however, ^152^Tb-PSMA-617 enabled acquisitions at late time points after injection and would, therefore, be of interest for pre-therapeutic dosimetry. Due to the challenging production, ^152^Tb is unlikely to be translated soon into broader routine clinical application. The presented study herein, however, is an important step towards the clinical application of other Tb radioisotopes in the near future.

## Additional file

Additional file 1: **Figure S1.** Representative chromatograms of (a) ^152^Tb-PSMA-617 and (b) ^177^Lu-PSMA-617 after successful radiolabeling. **Table S1.** Stability of ^152^Tb-PSMA-617. (DOCX 517 kb)

## Data Availability

All relevant data of this study are made available in this manuscript and in the Additional file 1 that has been provided.
