# Supplementary material for: Preclinical investigations and first-in-human application of 152Tb-PSMA-617 for PET/CT imaging of prostate cancer
Source: EJNMMI Res. 2019 Jul 25;9:68. doi: 10.1186/s13550-019-0538-1 (PMC6658632; doi:10.1186/s13550-019-0538-1)
Supplement: Supplementary file 1 — Figure S1. Representative chromatograms of (a) 152Tb-PSMA-617 and (b) 177Lu-PSMA-617 after successful radiolabeling. Table S1. Stability of 152Tb-PSMA-617. (DOCX 517 kb) [file 13550_2019_538_MOESM1_ESM.docx]

**SUPPLEMENTARY MATERIAL**

**Preclinical investigations and first-in-human application of ^152^Tb-PSMA-617**

Cristina Müller^1†^*, Aviral Singh^2,3†^, Christoph A. Umbricht^1^, Harshad R. Kulkarni^2^, Karl Johnston^4^, Martina Benešová^1^, Stefan Senftleben^2^, Dirk Müller^2^, Christiaan Vermeulen^1^, Roger Schibli^1,5^, Ulli Köster^6^, Nicholas P. van der Meulen^1,7^, Richard P. Baum^2^

^1^ Center for Radiopharmaceutical Sciences ETH-PSI-USZ, Paul Scherrer Institute, Villigen-PSI, Switzerland

^2^ Theranostics Center for Molecular Radiotherapy and Precision Oncology, ENETS Center of Excellence, Zentralklinik Bad Berka, Bad Berka, Germany

^3^ GROW - School for Oncology and Developmental Biology, Maastricht University, Maastricht, Netherlands

^4^ CERN, Geneva, Switzerland

^5^ Department of Chemistry and Applied Biosciences, ETH Zurich, Zurich, Switzerland

^6^ Institut Laue-Langevin, Grenoble, France

^7^ Laboratory of Radiochemistry, Paul Scherrer Institute, Villigen-PSI, Switzerland

^†^ contributed equally

**Corresponding author:**

PD Dr. Cristina Müller

Center for Radiopharmaceutical Sciences ETH-PSI-USZ

Paul Scherrer Institute

5232 Villigen-PSI

Switzerland

e-mail: cristina.mueller@psi.ch

phone: +41-56-310 44 54

fax: +41-56-310 28 49

**1. Quality Control and Stability Analysis of Radiolabeled Compounds**

Quality control of the radiolabeled compounds (^152^Tb-PSMA-617 and ^177^Lu-PSMA-617) was performed by HPLC (Fig. S1). A sample of the radioligand solution was diluted in sodium diethylenetriamine pentaacetic acid (Na-DTPA; 50 µM) and used for quality control (QC) by HPLC (Merck Hitachi LaChrom L-7100 HPLC pump coupled with a L-7200 autosampler, a D-7000 interface and an HPLC Radioactivity Monitor LB 506 B from Berthold) equipped with a reversed-phase column (Xterra^TM^, MS, C18, 5 µm, 150 x 4.6 mm; Waters). The mobile phase consisted of MilliQ water containing 0.1% trifluoroacetic acid (A) and acetonitrile (B). A gradient from 95% A and 5% B to 20% A and 80% B, over a period of 15 min, was used at a flow rate of 1.0 mL/min.

Stability analysis of ^152^Tb-PSMA-617 (10 MBq/nmol) was performed at room temperature at an activity concentration of 120 MBq in 3 mL phosphate-buffered saline (PBS). Samples were supplemented with and without 3 mg L-ascorbic acid as a scavenger to prevent radiolysis. Data on radiolytic stability was obtained at 2 h, 16 h, 40 h and 90 h post-radiolabeling on an aliquot of the radioligand solution using HPLC, in analogy to the quality control procedure (Table S1).


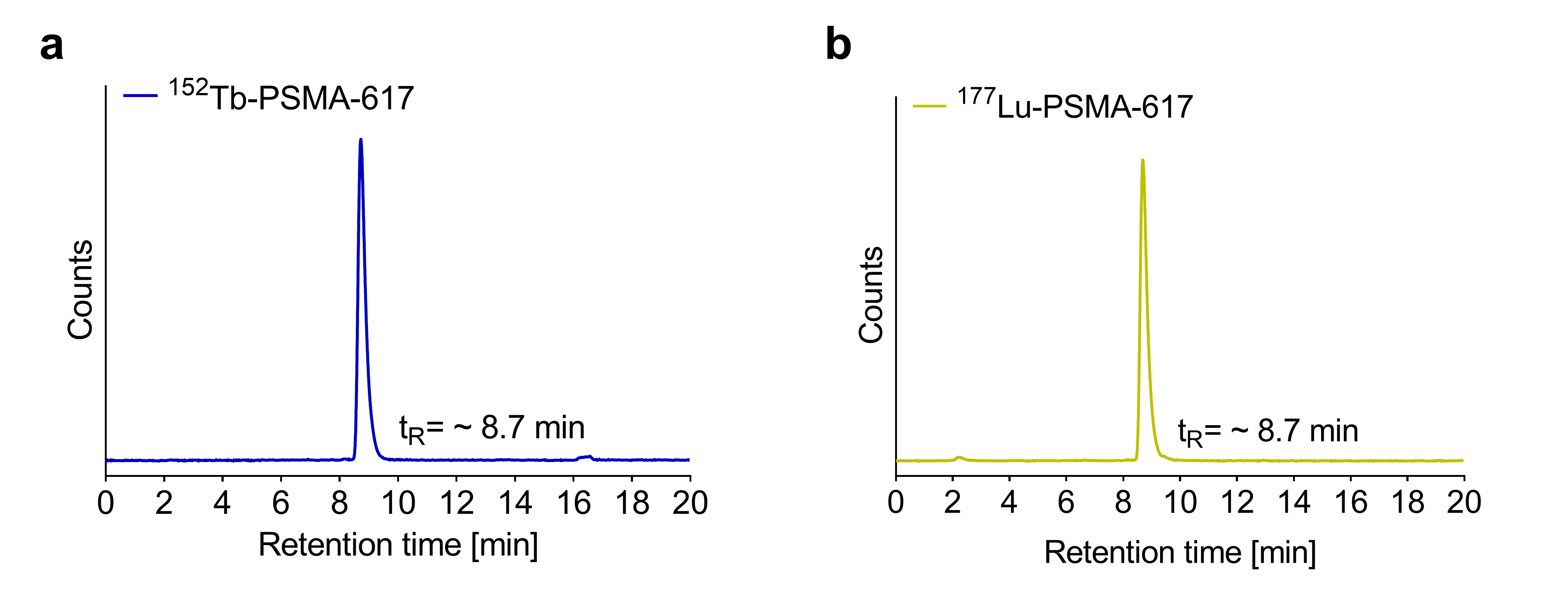


**Figure S1** Representative chromatograms of (a) ^152^Tb-PSMA-617 and (b) ^177^Lu-PSMA-617 after successful radiolabeling. Uncoordinated radiometals complexed with DTPA appear with a retention time of approximately t_R_ = 2 min.

**Table S1** Stability of ^152^Tb-PSMA-617

| **Incubation time**  [h] | **^152^Tb-PSMA-617** | |
| --- | --- | --- |
|  | **without L-ascorbic acid**  intact radioligand [%] | **L-ascorbic acid**  intact radioligand [%] |
| 2 | >98 | >98 |
| 16 | >95 | >98 |
| 40 | >95 | >98 |
| 90 | >95 | >98 |

**2. Cell Culture**

PC-3 PIP/flu cells, sublines of the androgen-independent PC-3 human prostate cancer cell line, originally derived from an advanced androgen-independent bone metastasis, were kindly provided by Prof. Dr. Martin Pomper (Johns Hopkins University, Baltimore, U.S.A.). These sublines were previously modified to express PSMA at high levels (PC-3 PIP cells), useful to test PSMA-targeting ligands [1-3]. A mock transfection yielded a PSMA-negative control cell line, which does not express PSMA (PC-3 flu cells). The cells were cultured in RPMI cell medium supplemented with 10% fetal calf serum, L-glutamine, antibiotics and puromycin (2 µg/mL) to maintain PSMA expression.

**3. Cell Internalization Study**

PC-3 PIP and PC-3 flu cells were seeded (3.0 x 10^5^ cells in 2 mL supplemented cell culture medium/well) in 12-well-plates, to allow adhesion and growth overnight. ^152^Tb-PSMA-617 and ^177^Lu-PSMA-617 were prepared at specific activities of 5 MBq/nmol. Cells were washed with phosphate buffered saline (PBS) and incubated with ^152^Tb-PSMA-617 (37.5 kBq, 3.75 pmol/well), or ^177^Lu-PSMA-617 (37.5 kBq, 3.75 pmol/well) at 37 °C for 1 h, 2 h, 4 h and 6 h, respectively. Total cell uptake was determined by washing the tumor cells twice with ice-cold PBS before lysis with NaOH (1 M, 1 mL), followed by transfer of the samples to RIA tubes to enable measurement of radioactivity in a γ-counter (Perkin Elmer, Wallac Wizard 1480). The cell internalized fraction of the radioligands was determined by washing the cells twice with PBS and once with ice-cold acidic stripping buffer (0.05 M glycine stripping buffer in 100 mM NaCl, pH 2.8) before cell lysis. The protein concentration was determined in each sample using a Micro BCA Protein Assay kit (Pierce, Therma Scientific). The results were expressed as percentage of total added radioactivity per 150 µg/mL protein.

**4. Preclinical PET/CT and SPECT/CT Imaging**

PET scans were performed with a small-animal bench-top PET scanner (G8, Perkin Elmer, U.S.A.). The energy window ranged from 150 keV to 650 keV. Static whole-body PET images were acquired for 10 min, followed by a CT scan of 1.5 min. The data were corrected for random coincidences, decay and dead time. The images were acquired using G8 acquisition software (version 2.0.0.10) and reconstructed with maximum-likelihood expectation maximization (MLEM).

SPECT/CT scans were performed using a four-headed, multiplexing, multi-pinhole small-animal SPECT camera (NanoSPECT/CT™, Mediso Medical Imaging Systems, Budapest, Hungary). Each head was fitted with a tungsten-based aperture of nine 1.4 mm-diameter pinholes and a thickness of 10 mm. The energy peaks of ^177^Lu were set at 56.1 keV (± 10 %), 112.9 keV (± 10 %) and 208.4 keV (± 10 %). The images were acquired using Nucline Software (version 1.02, Mediso Ltd., Budapest, Hungary). The real-time CT reconstruction used a cone-beam filtered backprojection. The reconstruction of SPECT data was performed using HiSPECT software (version 1.4.3049, Scivis GmbH, Göttingen, Germany).

**References**

1. Banerjee SR, Pullambhatla M, Byun Y, Nimmagadda S, Green G, Fox JJ, et al. ^68^Ga-labeled inhibitors of prostate-specific membrane antigen (PSMA) for imaging prostate cancer. J Med Chem. 2010;53:5333-41. doi:10.1021/jm100623e.

2. Banerjee SR, Pullambhatla M, Foss CA, Nimmagadda S, Ferdani R, Anderson CJ, et al. ^64^Cu-labeled inhibitors of prostate-specific membrane antigen for PET imaging of prostate cancer. J Med Chem. 2014;57:2657-69. doi:10.1021/jm401921j.

3. Umbricht CA, Benesova M, Schmid RM, Türler A, Schibli R, van der Meulen NP, et al. ^44^Sc-PSMA-617 for radiotheragnostics in tandem with ^177^Lu-PSMA-617-preclinical investigations in comparison with ^68^Ga-PSMA-11 and ^68^Ga-PSMA-617. EJNMMI Res. 2017;7:9. doi:10.1186/s13550-017-0257-4.
